# Assessment of Bone Metabolism in Male Patients With Benign Paroxysmal Positional Vertigo

**DOI:** 10.3389/fneur.2018.00742

**Published:** 2018-09-05

**Authors:** Yunqin Wu, Zhenyi Fan, Hang Jin, Qiongfeng Guan, Min Zhou, Xiaoxiong Lu, Li Li, Wang Yan, Chengyao Gu, Caijing Chen, Weiwei Han

**Affiliations:** ^1^Department of Neurology, Ningbo No. 2 Hospital, Ningbo, China; ^2^Department of Neurology, First Hospital of Jilin University, Changchun, China; ^3^Department of Rehabilitation, Ningbo No. 2 Hospital, Ningbo, China

**Keywords:** benign paroxysmal positional vertigo, bone mineral density, bone turnover markers, 25-hydroxyvitamin D, vitamin D deficiency

## Abstract

**Objective:** Several studies have suggested a probable association between benign paroxysmal positional vertigo (BPPV) and both reduction of bone mineral density (BMD) and serum vitamin D levels, but none of these studies have explored their findings by examining bone turnover markers (BTM) in male idiopathic BPPV patients. This study aimed to evaluate the relationship between BMD and serum 25-hydroxyvitamin D (25(OH) D), with the occurrence of BPPV along with the characteristics of bone metabolism in male idiopathic BPPV patients.

**Methods:** This retrospective study comprised 60 male idiopathic BPPV patients and 92 age-matched healthy controls referred to Ningbo No.2 Hospital during the period of February 2016 to February 2018. All subjects' serum levels of 25(OH) D, bone formation marker amino-terminal propeptide of type I procollagen (PINP), and bone resorption marker β-isomerized carboxy-terminal telopeptide of type I collagen (β-CTX) were measured. BMD was determined by dual energy X-ray absorption at the lumbar spine and hip.

**Results:** Among male patients with BPPV, the prevalence of BMD reduction was 35.0%, which was similar to that of 27.2% in healthy controls. There were significant differences in the mean serum 25(OH) D level and prevalence of vitamin D deficiency between the two groups, with *p*-values of 0.049 and 0.009, respectively. The bone turnover markers of PINP and β-CTX in BPPV patients were lower than those in healthy controls. Logistic regression showed that vitamin D deficiency were associated with BPPV with an odds ratio of 3.8 (95% confidence interval = 1.25–11.73).

**Conclusion:** Our study found that decreased serum vitamin D may be a risk factor for BPPV in male patients. The level of bone turnover among male patients with BPPV was lower than that among healthy controls.

## Introduction

Benign paroxysmal positional vertigo (BPPV) is one of the most common causes of vertigo, accounting for 36.5% of all dizziness complaints among the Chinese population (1). BPPV is characterized by transient vertigo, nausea and/or positional nystagmus provoked by head position change. Currently, the pathogenesis of BPPV is widely accepted; it is caused by the displaced otoconia floating into the semicircular canals or attaching to the cupula of the semicircular canals. BPPV can occur at any age, and the prevalence of BPPV rises rapidly with age (2, 3). The 1-year prevalence of BPPV attacks range from 0.5% in 18 to 39-year-olds to 3.4% in individuals over 60 years of age (3).

Canalith repositioning manoeuvers (CRM) provide a convenient and rapid method for the treatment of BPPV, which resolves the positional nystagmus in 80–100% of cases. Although CRM is very effective, nearly 44% of patients redevelop BPPV during 5 years of follow-up (4). Thus, BPPV has adverse consequences, including reduced ability to perform activities of daily living, increased psychosocial impact, and medical costs (5, 6). Various factors, such as head trauma, vestibular neuritis, Meniere's disease, and ear surgery, can induce BPPV attacks, but 50–70% of the underlying causes of otoconial degeneration and displacement from the utricle remain unknown (1, 4).

Multiple studies have suggested a relationship between BPPV, osteoporosis, and vitamin D deficiency (7–14). BPPV patients with lower bone mineral density (BMD) have an increased recurrence rate and require an increased number of CRM (8). Impressively, recent clinical studies have found that medications used to treat osteoporosis or vitamin D deficiency have a protective effect against BPPV (15–18). Because of the high incidence of BPPV in women, most previous studies selected women as study subjects. Thus, there are few reports assessing BMD and serum levels of 25-hydroxyvitamin D(25(OH) D) in male idiopathic BPPV patients, and the conclusions of these papers are contradictory(7, 9, 12, 19, 20). Moreover, none of these studies examined biochemical bone turnover markers (BTM) in male idiopathic BPPV patients.

Previously, work from our institution also identified that female de novo BPPV patients have significantly higher rates of reduction of BMD and vitamin D deficiency than general populations, and BPPV patients with low serum 25(OH)D levels show a significant increase in the recurrence rate (13, 14, 21). In this study, we aimed to investigate whether decreased BMD and/or vitamin D deficiency are also associated with the onset in male idiopathic BPPV patients, as well as to gather pilot data on bone metabolism in male idiopathic BPPV patients.

## Materials and methods

A total of 60 male patients, diagnosed with idiopathic BPPV were enrolled in the neurology department of Ningbo No. 2 hospital from February 2016 to 2018. BPPV diagnosis was based on a typical history of recurrent, brief positional vertigo and positive provocative manoeuvers. All BPPV patients were treated using the appropriate CRM according to the affected semicircular canal. For the detailed diagnosis and treatment methods, see elsewhere (1, 2). Another 92 age-matched healthy controls without history of vertigo or dizziness were recruited from the health check-up center of our hospital over the same period. Subjects were excluded in following condition:subjects who were uncooperative; had a history of parathyroid dysfunction;had a hip or lumbar spine fracture;had received vitamin D and calcium therapy, osteoporosis treatment, or corticosteroids and gonadotropin releasing hormone agonists therapy, which influence BMD or BTM; and those having secondary factors, such as a history of head trauma, vestibular neuritis, Meniere's disease, migraine, ear surgery, or sudden hearing loss, were excluded. To minimize the influence of confounding factors, we recorded all the data, including age, height, weight, ongoing health problems, medication history, and amount of physical activity as well as past and present of smoking and drinking.

All the procedures followed the tenets of the Declaration of Helsinki. This study has been approved by the Ethics Committee of Ningbo No. 2 Hospital (protocol number 2017-014-01), and written informed consent was obtained from all study subjects.

### Bone mineral densitometry

Dual-energy X-ray absorptiometry (GE Lunar Prodigy Scanner, GE Lunar Corporation, WI, USA) was used to measure BMD. The lowest T-score obtained from the lumbar vertebrae and femur levels was accepted as the valid T-score. The T-scores were expressed as standard deviations (*SD*) compared to the mean BMD of the young male population. BMD was defined according to the World Health Organization criteria; T-scores higher than −1.0 *SD* were considered normal, T-scores from −1.0 to −2.5 *SD* were considered to indicate osteopenia, and T scores lower than −2.5 *SD* were considered to indicate osteoporosis.

### Measurement of serum bone turnover marker

Fasting early morning venous blood was collected from all subjects for measurement of the following: 25(OH) D, amino-terminal propeptide of type I procollagen (PINP), and β-isomerized carboxy-terminal telopeptide of type I collagen (β-CTX). According to the internal standard, 25 (OH) D levels were classified as normal (>30 ng/ml), insufficient (20 to < 30 ng/ml), and deficient (< 20 ng/ml).

### Statistical analysis

The data were analyzed using SPSS 22.0 (SPSS Inc., Chicago, IL, USA). Quantitative values were expressed as the mean ± *SD*, and qualitative variables were described as numbers and percentages. Kolmogorov–Smirnov test was used to test the data distribution. The quantitative values such as: serum levels of 25(OH) D,PINP, and β-CTX were normally distributed. *T*-test, chi-square test or Fisher-test was used to determine the differences between the groups. Pearson's correlation analyses were used to assess the correlations between serum PINP and β-CTX, as well as other variables. Multiple logistic regression analysis was used to estimate the odds ratio (OR) for the association between BPPV and various factors. All *P*-values < 0.05 were considered statistically significant.

## Results

### Demographics and clinical characteristics of the subjects

A total 152 male subjects participated in this study, including 60 idiopathic BPPV patients and 92 healthy controls. The mean ages of BPPV patients and controls were 59.35 ± 13.24 years and 62.16 ± 10.58 years, respectively. A two-tailed *t*-test showed no significant difference between the two age distributions (*P* = 0.15;Table 1). Characteristics of the study population, including BMI, the proportion of diabetes mellitus or hypertension, history of smoking and drinking, and present history of smoking and drinking, are presented in Table 1.

**Table 1:** Clinical characteristics of benign paroxysmal positional vertigo patients and controls.

**Characteristics**	**Healthy controls (92)**	**BPPV (60)**	***P*-value**	**T/Z**
Age(years)	62.1 ± 10.6	59.4 ± 13.2	0.150	1.449
BMI(kg/cm^2^)	23.9 ± 2.8	23.6 ± 2.8	0.478	0.711
Diabetes (%)	16(17.4)	8(13.3)	0.502	0.45
Hypertension (%)	38(41.3)	23(38.3)	0.715	0.133
**SMOKING**				
Never smoking (%)	36(39.1)	26(43.3)	0.606	0.266
History of smoking (%)	11(11.9)	13(21.7)	0.109	2.575
Current smoking (%)	45(48.9)	21(35.0)	0.091	2.861
**DRINKING**				
Never drinking (%)	40(43.5)	28(46.7)	0.699	0.149
History of drinking (%)	9(9.8)	10(16.7)	0.210	1.573
Current drinking (%)	43(46.7)	22(36.7)	0.22	1.505
25(OH) D (ng/ml)	23.17 ± 6.49	20.99 ± 6.76	0.049	1.986
Insufficiency (%)	44(47.8)	21(35.0)	0.118	2.441
Deficiency (%)	31(33.7)	33(55.0)	0.009	6.762
**BMD**				
Normal (%)	67(72.8)	39(65.0)	0.305	1.054
Osteopenia (%)	20(21.7)	16(26.7)	0.485	0.488
Osteoporosis (%)	5(5.43)	5(8.33)	0.517^*^	—
PINP (ng/ml)	35.99 ± 13.19	32.74 ± 12.57	0.133	1.509
β-CTX (ng/ml)	0.39 ± 0.16	0.37 ± 0.19	0.605	0.519

*BMI, body mass index; 25(OH) D, 25-hydroxyvitamin D; BMD, bone mineral density; PINP, amino-terminal propeptide of type I procollagen; β-CTX, β-isomerized carboxy-terminal telopeptide of type I collagen*.

* *Fisher-test*.

### Comparison of BMD between groups

All sites with the mean T-score and BMD (g/cm^2^) value measured by dual-energy X-ray absorptiometry in BPPV patients were similar to those in healthy controls (all *P* > 0.05; Supplementary Table 1) except in the trochanter (*P* = 0.036; *P* = 0.023; Supplementary Table 1). In addition, regarding the proportion of reduction of BMD, osteoporosis was observed in 5 (8.33%), and osteopenia was observed in 16 (26.67%), which was similar to those in healthy controls (35.0 vs. 27.2%, *P* = 0.305; Table 1).

### Serum 25(OH) D, PINP, and β-CTX

The mean serum 25(OH) D levels in BPPV patients were significantly lower than in those healthy controls (20.99 ± 6.76 ng/ml vs. 23.17 ± 6.50 ng/ml, *P* = 0.049; Table 1). The prevalence of vitamin D deficiency was 55.0% (33/60) in BPPV patients, significantly higher than the 33.69% (31/92) of healthy controls (*P* = 0.009; Table 1). The mean PINP levels of BPPV patients was not significantly lower than those of the healthy controls, but the *P*-value was 0.133 (Table 1). The mean β-CTX levels were similar in the two groups (0.3737 ± 0.198 vs. 0.3891 ± 0.164, *P* = 0.605;Table 1).

There appeared to be a positive relationship between PINP and β-CTX in both groups, with higher PINP levels resulting in higher levels of β-CTX. The correlation coefficients ware 0.53 (*P* < 0.001) and 0.48 (*P* < 0.001) in the BPPV and healthy control groups, respectively (Table 2). PINP and β-CTX have a significant negative relationship with BMD T-score among healthy controls, with the correlation coefficients ware 0.53 (*P* < 0.001;Table 2) and 0.48 (*P* < 0.001;Table 2), respectively. In contrast, there was no such relationship among BPPV patients. A significant relationship between PINP and serum 25(OH) D levels in the BPPV group was identified, but the correlation coefficient was relatively low (*r* = −0.18, *P* = 0.0158;Table 2).

**Table 2:** Association between clinical parameters and biomarkers of bone turnover in two groups.

	**Healthy controls**				**BPPV**			
	**PINP**		β**-CTX**		**PINP**		β**-CTX**	
**Parameters**	***R***	***P*-value**	***R***	***P*-value**	***R***	***P*-value**	***R***	***P*-value**
Age(years)	−0.09	0.399	−0.02	0.843	−0.27	0.04	−0.23	0.083
BMI(kg/cm^2^)	−0.06	0.542	−0.24	0.022	0.09	0.461	0.048	0.717
25(OH) D (ng/mL)	−0.19	0.063	0.03	0.749	−0.18	0.0158	−0.04	0.786
BMD L1-L4 T (g/cm2)	−0.13	0.215	−0.25	0.018	−0.21	0.109	−0.18	0.166
BMD Total hip T (g/cm2)	−0.27	0.009	−0.34	0.001	−0.07	0.619	−0.23	0.082
PINP	–	–	0.48	< 0.001	–	–	0.53	< 0.001
β-CTX	0.48	< 0.001	–	–	0.53	< 0.001	–	–

*BMI: body mass index; 25(OH) D, 25-hydroxyvitamin D; BMD, bone mineral density; PINP, amino-terminal propeptide of type I procollagen; β-CTX, carboxy-terminal telopeptide of type I collagen*.

### Multiple logistic regression analysis

Multiple logistic regression analyses, adjusted for age, BMI, P1NP, β-CTX, the presence of diabetes, hypertension, and BMD, demonstrated that 25(OH) D deficiency was associated with BPPV with ORs of 3.8 (95% CI = 1.25–11.73, *P* = 0.019; Table 3).

**Table 3:** Logistic regression analysis of the potential link between BPPV and variables.

**Variables**	**Odds ratio**	***P*-value**	**95%CI**
25-hydroxyvitamin D			
Deficiency	3.8	0.019	1.25–11.73

*BPPV, benign paroxysmal positional vertigo; CI, confidence interval*.

## Discussion

As the first report of bone metabolism in male patients with idiopathic BPPV, this study found that serum 25(OH) D levels in male patients with idiopathic BPPV were significantly lower than those in the healthy controls, and low 25(OH) D levels were associated with an increased BPPV occurrence. In addition, this study provides indications of *l*ower bone turn-over rates in BPPV patients compared with healthy controls.

Otoconia, such as bone, are a result of inorganic calcium carbonate (calcium phosphate in bone) forming an orderly deposition onto a framework of organic matrix (22). The inner core of the organic matrix of otoconia is made up of glycoproteins and proteoglycans in which otoconin 90 is the main protein, and the outer surface consists mostly of calcium carbonate. How otoconia are formed and subsequently embedded in the otoconial membrane remains unclear. Although it is still unclear whether mature otoconia are in the dynamic turnover process, they are easily influenced by various factors (23, 24). Otoconia had loose morphology and were occasionally displaced into the semicircular canals in otoconin 90-null mice (25). Otoconin 90 expression levels were decreased in bilateral ovariectomy rats, while estrogen replacement reversed the decrease of otoconin 90 levels (26). Furthermore, bilaterally ovariectomised mice have significant balance-related behavioral deficits and that estrogen deficiency compromises otoconia maintenance and anchoring by reducing the expression of the otoconial component and anchoring proteins (27). Distinctive changes in the morphometry and distributional pattern of otoconia also have been commonly observed in aged rats (28, 29).

Previously, some studies have investigated BMD and/or serum 25(OH) D levels in male patients with BPPV, but the conclusions were inconsistent. Jeong et al. (7). measured the BMD of 67 male idiopathic BPPV patients, and found 43.4% had osteopenia and 11.9% had osteoporosis, which was significantly higher than in the controls. In addition, Jeong et al. (9). reported that serum 25(OH) D levels were lower in 37 male patients with BPPV than in controls. While, a retrospective study including 30 male patients with BPPV revealed that serum 25(OH) D concentrations were significantly decreased compared with controls, whereas BMD results showed no significant differences (12). Different from previous findings, there were no significant differences in mean T-scores and vitamin D levels, osteoporosis, and vitamin D deficiency prevalence between 29 male BPPV patients and controls (19). Similarly, Cikrikc isk et al. conducted a study only including 17 male BPPV patients and did not find that vitamin D deficiency was associated with BPPV incidence or recurrence (20).

Due to multiple different factors, such as the size limitation, racial background, distribution of age, time of follow up, seasonal factors, nutrition and lifestyle habits in these studies, the conclusions of BPPV between low BMD and vitamin D deficiency were paradoxical. Serum vitamin D levels fluctuate with the seasons, which reach lowest level during the period of March to May, and reach highest level during the period of September to October in the northern hemisphere (30, 31). Therefore, the time of the blood draws to measure vitamin D level to BPPV episode is crucial. We should be aware that a person with an “optimal” level of vitamin D in the summer may well become “deficient” in the winter without any change in diet and as a result of changes in sun exposure.

To minimize the effects of seasonal factors and geographic location on serum vitamin D levels, we consecutively selected male idiopathic BPPV patients and healthy controls from the local community during the period from February 2016 to 2018. Possible influencing factors, such as age, BMI, lifestyle and comorbidities, were similar in the groups. We found that decreased 25(OH)D may be associated with BPPV occurrence, while the reduction of BMD was not corrected with BPPV occurrence.

Dual-energy X-ray absorptiometry measures BMD and remains the gold standard for diagnosing osteoporosis. However, bone loss is slower in men than in women, especially before the age of 70. The rate of bone loss may vary according to the skeletal site. In particular, lumbar spine is not a reliable skeletal site for estimation of bone loss in men because of frequent osteoarthritis (32). BMD was not an early indicator of changes in the bones and dose not signify the current rate of bone loss. Therefore, there is an obvious temporal incongruence with BPPV episodes, which tend to present abruptly (33). Therefore, much interest has been focused on BTM that can provide better assessments of bone dynamic changes (34). Nevertheless, only three studies have examined serum markers of bone turnover in female BPPV patients. Ko et al. reported that female idiopathic BPPV patients had higher proportions of elevated urinary deoxypyridinoline than did controls (35). Parham et al reported that postmenopausal women with idiopathic BPPV had higher P1NP levels compared to age-matched osteoporotic women, while mean serum β-CTX, vitamin D, and Ca^2+^ levels were similar between the two groups (36). In a similar vein, Lee et al reported that only osteocalcin and urinary deoxypyridinoline levels were significantly elevated in osteoporotic patients with BPPV, and the serum alkaline phosphatase and β-CTX did not demonstrate any significant differences among these groups (37).

PINP and β-CTX were recommended by the International Osteoporosis Foundation as the standard bone formation and resorption markers in the management of osteoporosis (34). Good agreement of age-related changes of PINP and β-CTX is observed in men between 20 and 60 years of age. In contrast, data on serum PINP and β-CTX level in elderly men are discordant(32). A large-scale, multi-center, cross-sectional study conducted in China found high levels of PINP and β-CTX in men during late adolescence, remained at low levels from 40 to 69 years of age, and then declined further after age 70 (38). In our preliminary study, we found that the levels of PINP and β-CTX in BPPV patients were lower than those in the healthy controls, notably the *p*-value of the difference in the PINP between the groups was only 0.013. Lack of statistical significance of PINP and β-CTX can be partly determined by a small number of investigated subjects and/or by a limited age range. But, such a change maybe potentially associated with the dysfunction of osteoblasts and osteoclasts and adversely affected calcium metabolism in the BPPV patients.

BPPV is a benign disease, and CRM is very effective, but a number of BPPV patients may suffer recurrences. Thus far, we have not had access to the inner ear in real-time nor an appropriate animal model to study the metabolic processes of otoconia. As a consequence, we are limited to clinical observation of symptomatic BPPV patients and management of individual BPPV episodes with CRM. Recently, studies have found that supplementation with vitamin D or treated osteoporosis may prevent BPPV recurrences. BPPV is so common, and the recurrence rate is so high that, even if the supplementation with vitamin D or treated osteoporosis inhibits recurrence only in a small percentage of cases, this means a large number of cases with improvement. Despite differences between bone and otoconia metabolism, more studies have confirmed the association between BPPV and disorders of bone turnover, implying otoconia are subject to the same systemic regulatory mechanisms whose disruption leads to the reduction of BMD. Thus, analyzing bone metabolism in BPPV patients would be the first step in developing treatment strategies to reduce the incidence of recurrent BPPV.

Our study had several limitations. First, this is a single-center retrospective study performed in a local institution with a relatively small sample size, and the participants do not represent the entire population of Ningbo. Second, we did not measure other markers for bone metabolism and probable markers for otoconia. Recent reports suggested that an inner ear collagen named otolin-1 could be a serological biomarker for otoconia degeneration (39–41). In further studies, the correlations between otolin-1 and bone metabolism markers were explored. Finally, our study did not include a dynamic assessment of the changes in bone metabolism and 25(OH) D in BPPV patients. Thus, we cannot ruled the effects of seasonal factors on vitamin D and BTM. More prospective studies with the dynamic assessment of changes in bone metabolism are needed to clarify these issues, possibly including the use of animal models to provide more information for the study of the potential pathogenesis of otoconia degeneration.

Although there are some limitations, this is the first study with a relatively large sample of male BPPV patients as the study subjects. In this study, we found that decreased 25(OH) D may be associated with BPPV occurrence, and bone turnover level in male BPPV patients was lower than that in the healthy controls.

## Conclusions

In summary, this study demonstrated that low levels of 25(OH) D may be associated with BPPV occurrence, and low bone turnover rates in BPPV patients support the connection between BPPV and disturbance of bone turnover.

## Author contributions

YW, ZF, and WH conceived and led the work. YW, WH, MZ, HJ, XL, LL, QG, WY, CG, and CC reviewed and acquired the patients' medical record. WY, CG, HJ, and MZ performed the statistical analysis of the data. YW, WH, and ZF drafting and revising the manuscript with input from all co-authors.

### Conflict of interest statement

The authors declare that the research was conducted in the absence of any commercial or financial relationships that could be construed as a potential conflict of interest.
