# Supplementary material for: Assessment of Bone Metabolism in Male Patients With Benign Paroxysmal Positional Vertigo
Source: Front Neurol. 2018 Sep 5;9:742. doi: 10.3389/fneur.2018.00742 (PMC6135048; doi:10.3389/fneur.2018.00742)
Supplement: Supplementary file 1 [file Table_1.doc]

**Supplementary Table 1.** T-scores and BMD (g/cm^2^) in the control and BPPV groups.

|  |  |  | Healthy controls (92) | BPPV (60） | P-value | T-value |
| --- | --- | --- | --- | --- | --- | --- |
| Lumbar | L1 | T-score | -0.003 ± 1.48 | -0.07 ± 1.66 | 0.791 | 0.265 |
|  |  | BMD, g/cm^2^ | 1.023 ± 0.178 | 1.016 ± 0.195 | 0.797 | 0.257 |
|  | L2 | T-score | 0.016 ± 1.71 | -0.035 ± 1.54 | 0.847 | 0.193 |
|  |  | BMD, g/cm^2^ | 1.098 ± 0.203 | 1.092 ± 0.177 | 0.851 | 0.188 |
|  | L3 | T-score | 0.475 ± 1.74 | 0.298 ± 1.66 | 0.624 | 0.534 |
|  |  | BMD, g/cm^2^ | 1.174 ± 0.206 | 1.154 ± 0.194 | 0.613 | 0.541 |
|  | L4 | T-score | 0.821 ± 1.68 | 0.3 ± 1.79 | 0.072 | 1.813 |
|  |  | BMD, g/cm^2^ | 1.198 ± 0.204 | 1.136 ± 0.208 | 0.072 | 1.815 |
|  | L1-L4 | T-score | 0.348 ± 1.54 | 0.093 ± 1.59 | 0.327 | 0.983 |
|  |  | BMD, g/cm^2^ | 1.128 ± 0.186 | 1.101 ± 0.183 | 0.367 | 0.904 |
| Femur | Neck | T-score | -0.332±1.09 | -0.495±1.09 | 0.368 | 0.903 |
|  |  | BMD, g/cm^2^ | 0.939±0.140 | 0.915±0.142 | 0.281 | 1.082 |
|  | Word’s triangle | T-score | -1.009±1.06 | -1.120±1.06 | 0.527 | 0.634 |
|  |  | BMD, g/cm^2^ | 0.736±0.155 | 0.719±0.157 | 0.501 | 0.674 |
|  | Trochanter | T-score | -0.016±1.02 | -0.392±1.14 | **0.036** | 2.114 |
|  |  | BMD, g/cm^2^ | 0.832±0.123 | 0.784±0.134 | **0.023** | 2.291 |
|  | Total | T-score | 0.027±1.03 | -0.163±1.23 | 0.305 | 1.029 |
|  |  | BMD, g/cm^2^ | 1.000±0.135 | 0.966±0.153 | 0.153 | 1.435 |

*BMD, bone mineral density; Bold face indicates P < 0.05.*
